# To Contributors

**Published:** 1884-07

**Authors:** 


### Editorial.

### TO CONTRIBUTORS.

This number of the INDEPENDENT PRACTITIONER has been enlarged to sixty-four pages. Notwithstanding this, considerable matter that should have been published in it, and which we are as desirous to present as any one can be to see, is inexorably crowded out. Each month we pack in all that it is possible to get between the covers, and each month desirable articles must lie over. In this number there should have appeared reports of the Chicago Dental Society, of the Central Association of Northern New Jersey, and of the Northern Ohio Dental Association, all of them excellent reports, besides a number of good articles, some of which were sent in at our personal solicitation. Thanks, kind friends, for your appreciative favors. They shall see the light at the earliest possible moment. We do not care that editorial and book notices are excluded; we have but few briny tears to shed when our associates are shut out; we can submit with fortitude to a knowledge of the fact that extracts and stale matter of all kinds can find no place; but it is a source of sorrow when desirable and instructive communications must wait their turn. Help the journal to enough of subscribers to warrant the outlay, and its size shall be made sufficient to contain everything desirable. But none of our syndicate has any private business to boom, nor any personal axe to grind, and, therefore, our outlay must in a measure be limited by what we receive.

